# An excimer lamp to provide far-ultraviolet C irradiation for dining-table disinfection

**DOI:** 10.1038/s41598-023-27380-2

**Published:** 2023-01-07

**Authors:** Mengqiang Lv, Jin Huang, Haofu Chen, Tengfei (Tim) Zhang

**Affiliations:** 1 Tianjin Laboratory of Indoor Air Environmental Quality Control, School of Environmental Science and Engineering, Tianjin University, Tianjin, China; 2 School of Civil Engineering, Dalian University of Technology, Dalian, China

**Keywords:** Environmental impact, Civil engineering

## Abstract

Dining tables may present a risk to diners by transmitting bacteria and/or viruses. Currently, there is a lack of an environmental-friendly and convenient means to protect diners when they are sitting together. This investigation constructed far-UVC excimer lamps to disinfect dining-table surfaces. The lamps were mounted at different heights and orientations, and the irradiance on table surfaces was measured. The irradiation doses to obtain different inactivation efficiencies for *Escherichia coli* (*E. coli*) were provided. In addition, numerical modeling was conducted for irradiance and the resulting inactivation efficiency. The surface-to-surface (S2S) model was validated with the measured irradiance. The germicidal performance of far-UVC irradiation, the far-UVC doses to which diners were exposed, and the risk of exposure to the generated ozone were evaluated. The results revealed that an irradiation dose of 12.8 mJ/cm^2^ can disinfect 99.9% of *E. coli* on surfaces. By varying the lamp irradiance output, the number and positions of the lamps, the far-UVC irradiation can achieve a 3-log reduction for a dining duration of 5 min. Besides, the far-UVC lamp has a low damage risk to diners when achieving an effective inactivation rate. Moreover, there is virtually no ozone exposure risk in a mechanically ventilated dining hall.

## Introduction

Dining tables are easily contaminated by microbes^1,2^. Microbes on dining table surfaces can be either bacteria or viruses^3,4^. The bacteria can be transmitted to dining-table surfaces by (1) wiping tables with polluted rags and sponges, (2) deposition of airborne microorganisms, and (3) contacting with tainted food^5–7^. Bacteria on dining-table surfaces can survive for hours or even weeks^8^. The viruses may settle on dining tables with the droplets released from infected diners^9^. Even when separated from the host cell, the virus may remain alive on dining-table surfaces for 2–7 days under suitable conditions^4^. Disinfection of microbes on dining tables can be an effective way to minimize the resulting human infection via the surface touch route.

Chemical disinfectants have long been used to kill microbes on surfaces. Dining-table disinfectants include chlorinated solvents, ethyl alcohol, peroxyacetic acid, and quaternary ammonium salt solvent^10^, etc. These disinfectants inactivate microbes either by denaturing the biological proteins through oxidation or by accelerating water loss from biological bodies under the force of surface tension^11,12^. Chemical disinfectants are quite effective in killing the vast majority of microbes^13^. However, most chemical disinfectants are not environmentally friendly. More critically, chemical disinfectants can be harmful to human health if exposure exceeds the allowed upper limit. The reported respiratory and skin symptoms due to overexposure to residual chlorinated disinfectants during the Ebola epidemic in 2014 underscore the shortcomings of chemical disinfection^14^. Hence, chemical disinfection should be used with caution on dining tables.

Ultraviolet C (UVC) radiation is also quite effective for inactivating microbes^15^, and no residue is produced on surfaces. The traditional UVC with a wavelength of 254 nm is commonly generated by an electric arc through vaporized mercury. The emitted photons can deeply penetrate microbial bodies and destroy either ribonucleic acid (RNA) or deoxyribonucleic acid (DNA)^16^. Thus, both replication and proliferation of the irradiated microbes are prevented. However, UVC_254nm_ cannot be safely used to irradiate the human body. Conjunctivitis, erythema, and even skin cancer can result from excessive UVC_254nm_ exposure^17^. Hence, UVC_254nm_ disinfection during dining may be prohibited.

In recent years, far-UVC with wavelengths ranging from 207 to 222 nm has received great attention^18^. Far-UVC rays can be generated by an excimer lamp inside which the noble gas is ionized by a high voltage. Far-UVC reportedly has a similar germicidal effect on bacterial cells and viruses and is more potent in killing bacterial endospores than UVC_254nm_^19^. Notably, the penetration depth of far-UVC rays in human cells is very limited because the emitted photons are preferentially absorbed by the corneum and then assimilated by cytoplasmic proteins^20^. Thus, the high-energy photons are prevented from entering the nucleus. Therefore, far-UVC seems suitable for disinfection in the presence of humans.

The ionization for far-UVC rays may recombine oxygen atoms in the air and generate ozone. The ozone emission rates of excimer lamps (for far-UVC) are much lower than those of the conventional mercury lamps (mainly for UVC_254nm_) with the same input power. However, the indoor ozone concentration may still rapidly rise if excimer lamps are used in a volume-limited dining room with insufficient ventilation. Long-term exposure to high concentrations of ozone can lead to numerous adverse health effects^21^. Consequently, the possible ozone exposure when adopting far-UVC irradiation for disinfection must also be evaluated.

The above review revealed that far-UVC may prevail over chemical disinfectants and traditional UVC_254nm_ for disinfection of frequently touched surfaces. Contaminated dining tables may contribute to microbe transmission. To date, there is a lack of an environmental-friendly and convenient method that can be long used to protect diners when they are sitting together. This investigation conducted both experiments and numerical modeling to fill this knowledge gap.

## Results

This section presents the germicidal performance of far-UVC irradiation, far-UVC doses to which diners are exposed, and the possible risk of exposure to the associated ozone.

### Measured irradiance on a dining table and numerical model validation

**Figure 1:**
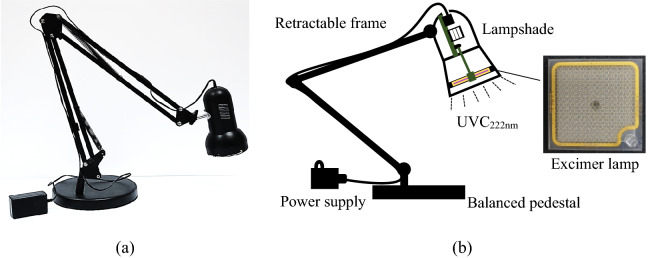
A far-UVC lamp constructed for dining-table disinfection: (**a**) photograph of the exterior appearance; (**b**) diagram of the structure and photograph of an excimer lamp.

**Figure 2:**
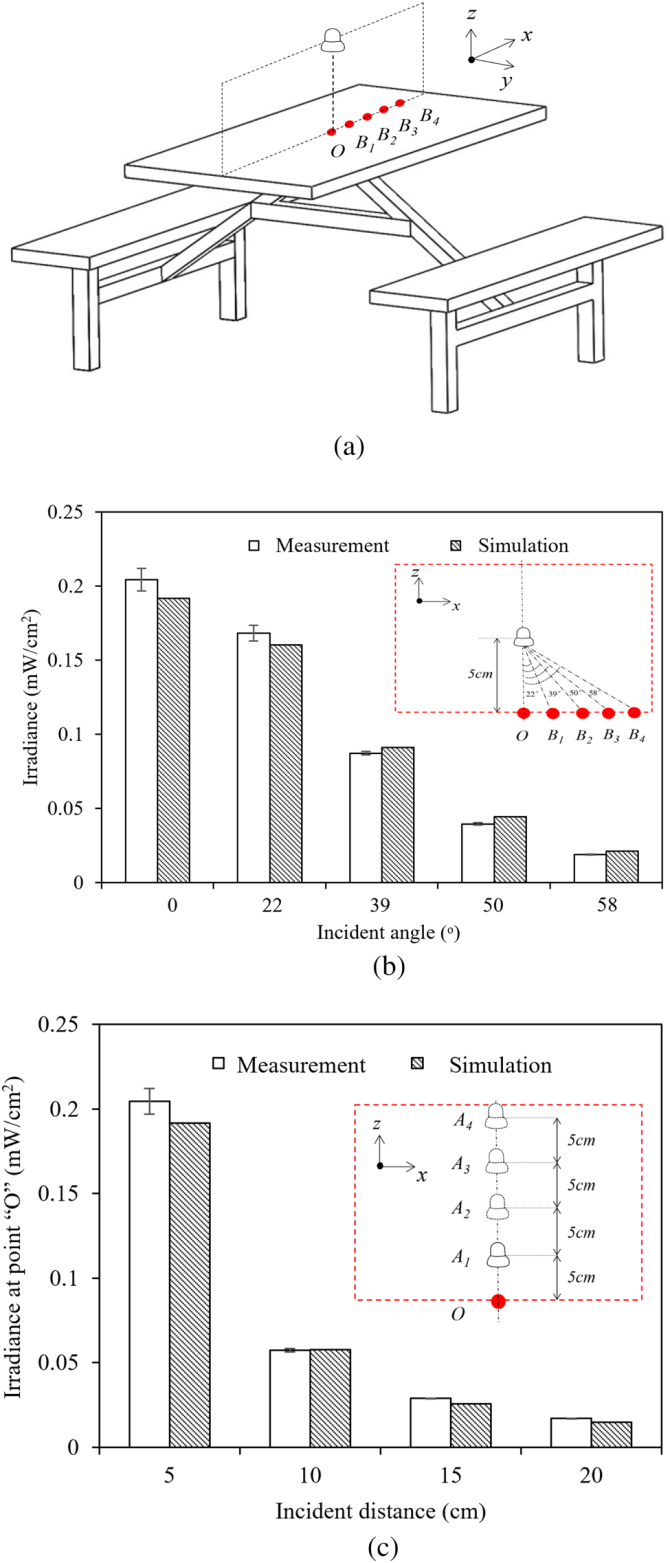
Comparison between the predicted and measured irradiance on the dining-table surface: (**a**) Schematics of far-UVC irradiance sampling positions; (**b**) irradiance versus incident angle; (**c**) irradiance versus incident distance.

Figure 1 presents the constructed far-UVC lamp for dining-table disinfection, modified from a market-available desk lamp, by replacing the existing lamp for illumination with the excimer lamp (Eden Park, USA). Variation of the table surface irradiance with the incident angle and incident distance is shown in Fig. 2. When the incident distance was maintained at a constant value of 5 cm, the irradiance exhibited a decay pattern with the incident angle, as shown in Fig. 2b. The irradiance reached a peak at the vertical irradiation and decreased to 0.17 mW/cm^2^, 0.19 mW/cm^2^, 0.05 mW/cm^2^, and 0.03 mW/cm^2^ when the incident angles were increased to 22°, 39°, 50°, and 58°, respectively, in accordance with Lambert’s law. When the incident angle was maintained at 0°, the irradiance decreased with the incident distance, as shown in Fig. 2c. The irradiance was 0.21 mW/cm^2^ at an incident distance of 5 cm and decreased to 0.06 mW/cm^2^, 0.03 mW/cm^2^, and 0.02 mW/cm^2^ when the incident distance was increased to 10 cm, 15 cm, and 20 cm, respectively, in accordance with the variation of the view factor. The surface-to-surface model (S2S)^22^ not only successfully predicted the variation of irradiance with incident distance and incident angle but also obtained a relative deviation of less than 15% as compared with the measurement, indicating that the numerical modeling was highly accurate.

### Relationship between inactivation efficiency and irradiation dose

**Figure 3:**
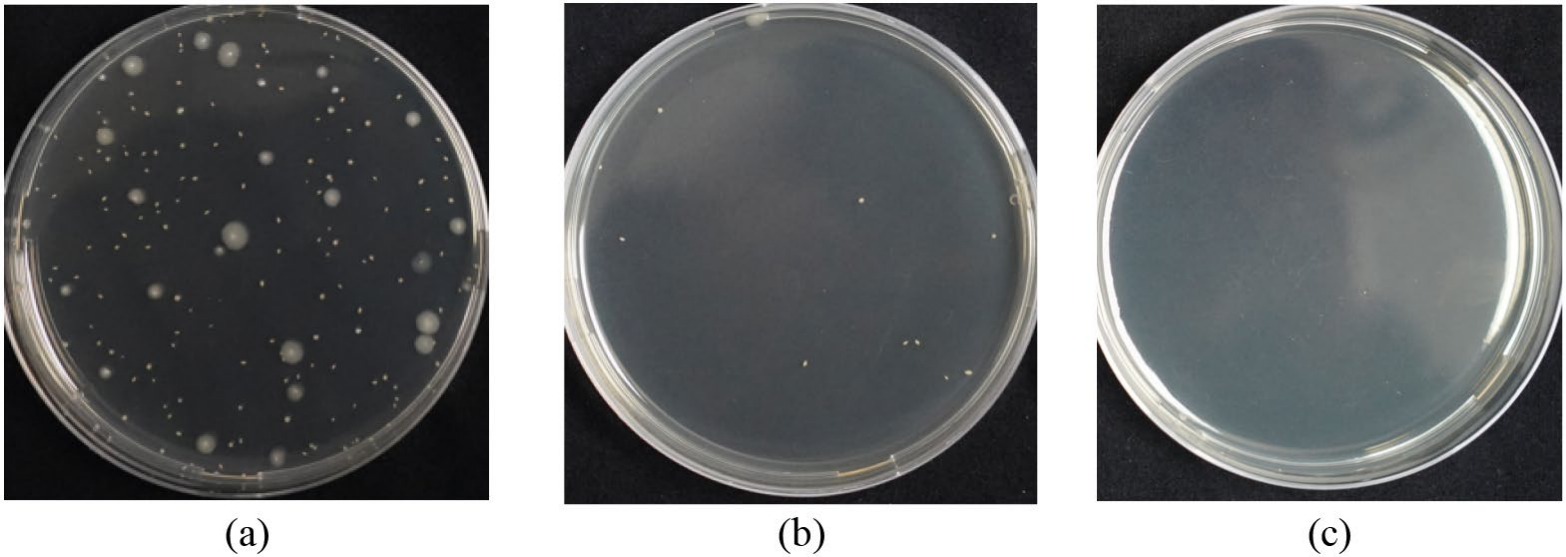
Counted live *E. coli* under an irradiation dose of 1 mJ/cm^2^ with the pouring plate method: (**a**) the sample with the inoculated *E. coli* before far-UVC irradiation; (**b**) the sample with the inoculated *E. coli* after irradiation; (**c**) the sample without inoculation.

*Escherichia coli* (*E. coli*) were irradiated by the far-UVC lamp, and the numbers of live *E. coli* under different irradiation doses were measured. Taking an irradiation dose of 1 mJ/cm^2^ as an example, the live *E. coli* before and after disinfection are exhibited in Fig. 3. Before the far-UVC irradiation, abundant *E. coli* was present as shown in Fig. 3a. After irradiation, 92.3% of *E. coli* was inactivated, and only a small percentage was still alive, as shown in Fig. 3b. No microorganism was found in the test sample that had not been inoculated with *E. coli*, as shown in Fig. 3c, indicating that the samples were not polluted. The results indicate that far-UVC performs well in reducing *E. coli*.

**Figure 4:**
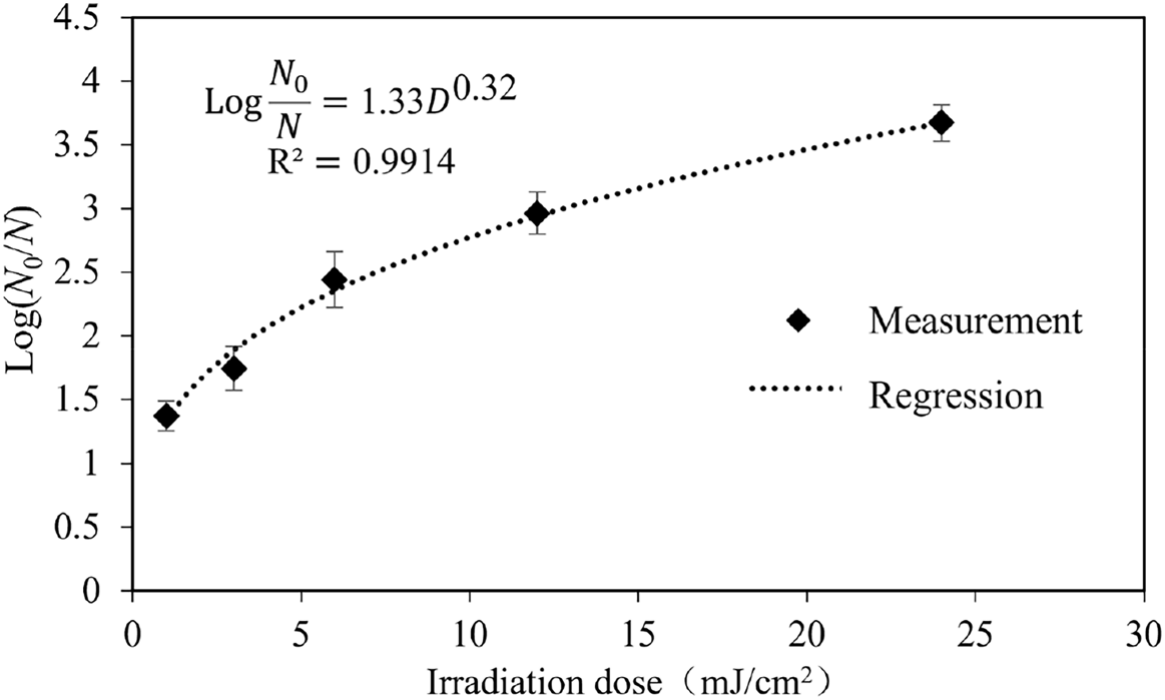
Relationship between inactivation efficiency and irradiation dose for *E. coli*.

As shown in Fig. 4, the log reduction of *E. coli* generally increased with the irradiation dose. Under irradiation doses of 1 mJ/cm^2^, 3 mJ/cm^2^, 6 mJ/cm^2^, 12 mJ/cm^2^, and 24 mJ/cm^2^, the log reductions were 1.37, 1.74, 2.44, 2.96, and 3.67, respectively. The log reduction increases with the irradiation dose exponentially based on Hom’s model^23^. If the target is an inactivation efficiency of no less than 3-log, then the required irradiation dose should be at least 12.8 mJ/cm^2^.

### Numerically predicted performance of the far-UVC lamps in a dining hall

#### Inactivation efficiency on the dining-table surface

**Figure 5:**
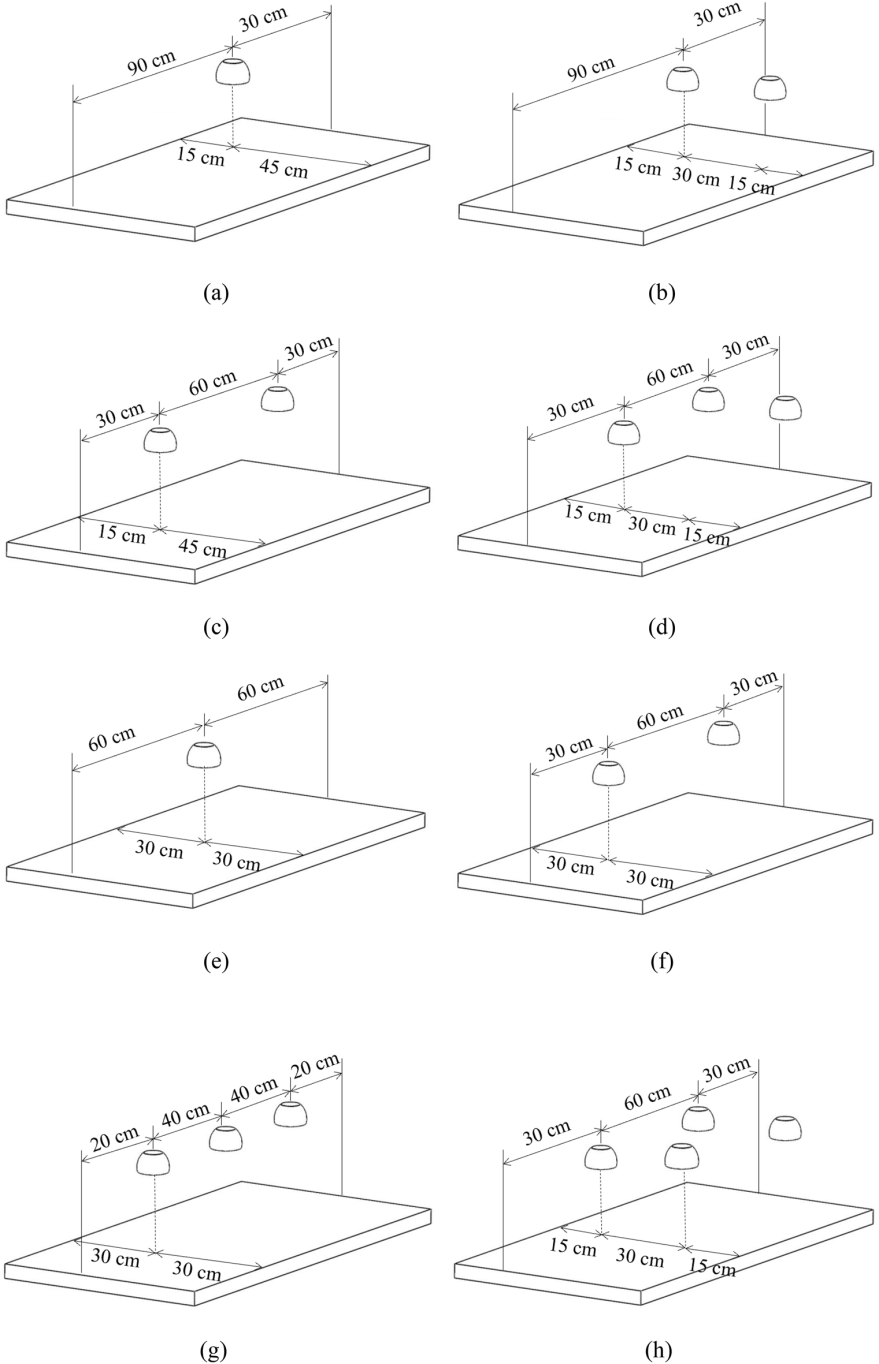
Required numbers and possible positions of far-UVC lamps for typical dining situations: (**a**) one lamp for a single diner; (**b**) two lamps for two diners sitting face to face; (**c**) two lamps for two diners sitting on the same side; (**d**) three lamps for three diners; (**e**) one lamp for four diners; (**f**) two lamps for four diners; (**g**) three lamps for four diners; and (**h**) four lamps for four diners.

Figure 5 exhibits the eight typical dining situations. Among these, strategy (e) with “one lamp for four diners” was rejected, because an inactivation efficiency of 3-log could not be achieved with lamp surface irradiance less than 100 mW/cm^2^. The current excimer lamp may provide a peak surface irradiance as high as 100 mW/cm^2^. This finding implies that a single lamp was not sufficient for disinfecting the whole dining-table surface under an incident distance of 25 cm.

**Figure 6:**
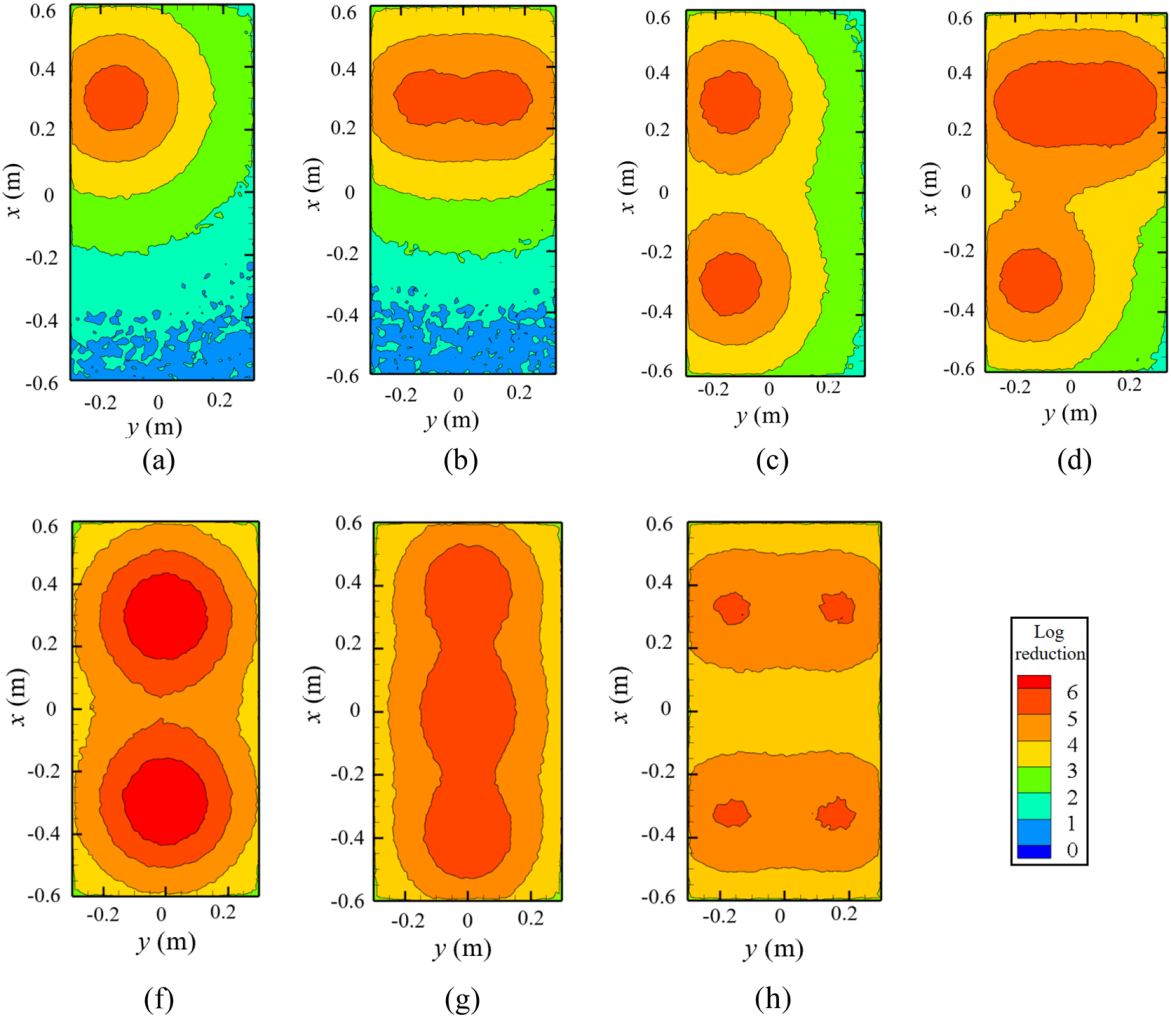
Numerically modeled inactivation efficiency distribution for different dining situations: (**a**) one lamp for a single diner; (**b**) two lamps for two diners sitting face to face; (**c**) two lamps for two diners sitting on the same side; (**d**) three lamps for three diners; (**f**) two lamps for four diners; (**g**) three lamps for four diners; and (**h**) four lamps for four diners.

Figure 6 presents the modeled distribution of inactivation efficiency on tables, with a dining duration of 5 min. Notably, the required lamp surface irradiance varied with the situation. Table 1 presents the minimum required lamp surface irradiance to achieve an inactivation efficiency of no less than 3-log in the dining area. The inactivation efficiency reached maximum values under the lamps and decreased as the distance from the lamp increased. For the situation with a single lamp, the inactivation efficiency isolines were circularly distributed on the dining-table surface as shown in Fig. 6a. When a larger number of lamps were used, the overlap region would exhibit a higher inactivation efficiency, as displayed in Fig. 6b–d and Fig. 6f–h.

**Table 1:** Minimum irradiance required for far-UVC lamps under different dining situations.

Situation	Number of diners	Sitting pattern	Far-UVC lamp number	Minimum lamp surface irradiance (mW/cm^2^)
(a)	1	–	1	42
(b)	2	Face to face	2	33 + 33
(c)	2	Same side	2	42 + 42
(d)	3	–	3	33 + 33 + 42
(f)	4	–	2	90 + 90
(g)	4	–	3	48 + 48 + 48
(h)	4	–	4	30 + 30 + 30 + 30

As shown in Table 1, for one diner with a single lamp, at least 42 mW/cm^2^ irradiance at the lamp surface was required to ensure an inactivation efficiency of 3-log for one quarter of the dining table within 5 min. For two diners sitting face to face, the irradiance of no less than 33 mW/cm^2^ was needed for each lamp. This value was smaller than that in situation (a) because of the overlap of irradiating regions. For two diners sitting on the same side, each lamp had to irradiate a distinct dining area due to the very limited overlapping zone. Consequently, the required irradiance was identical to that in situation (a). From the perspective of energy saving, sitting face to face is recommended for two diners. The situation of “three lamps for three diners” was like a combination of situation (a) and situation (b). For the situation with four diners, when two, three or four lamps were used, the minimum irradiance required for each lamp was 90 mW/cm^2^, 48 mW/cm^2^, or 30 mW/cm^2^, respectively. If the total energy input is a concern, four lamps are preferrable. In addition, more lamps mean greater flexibility for the users but will entail a larger initial investment.

#### Far-UVC irradiation doses received by diners

**Figure 7:**
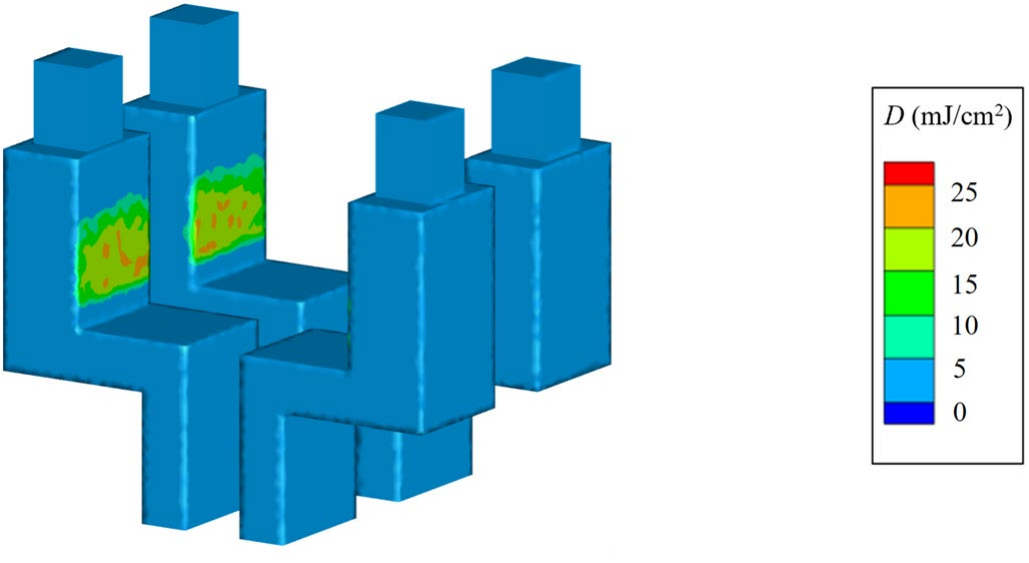
Far-UVC irradiation dose received by diners in the situation of “two far-UVC lamps for four diners”.

Dining situation (f), which had the highest total irradiance, was selected as an example for analysis of the far-UVC exposure to diners. The far-UVC irradiation received by diners in 5 min is shown in Fig. 7. The diners’ eyes were not irradiated at all due to the relatively low height of the lamps, and also due to the protection provided by the lampshade. The high dose of the far-UVC irradiation was mainly concentrated in the abdomen, with a peak value of 23 mJ/cm^2^. Far-UVC exposure to the human surface did not exceed the 8-h daily threshold limit values (TLV) of 23 mJ/cm^2^ proposed by the International Commission on Non-Ionizing Radiation Protection (ICNIRP)^24^. Exposure doses for the other six situations were also evaluated. None of the diners’ eyes were exposed to far-UVC in these cases. Moreover, the maximum doses to which the human surfaces were exposed were all less than 20 mJ/cm^2^. The abdomen is commonly covered by clothing. Consequently, the received far-UVC exposure would be lower than the above-analyzed values. Meanwhile, the shape of the lampshade can also be optimized to reduce the irradiation on human surfaces.

#### Ozone exposure

**Figure 8:**
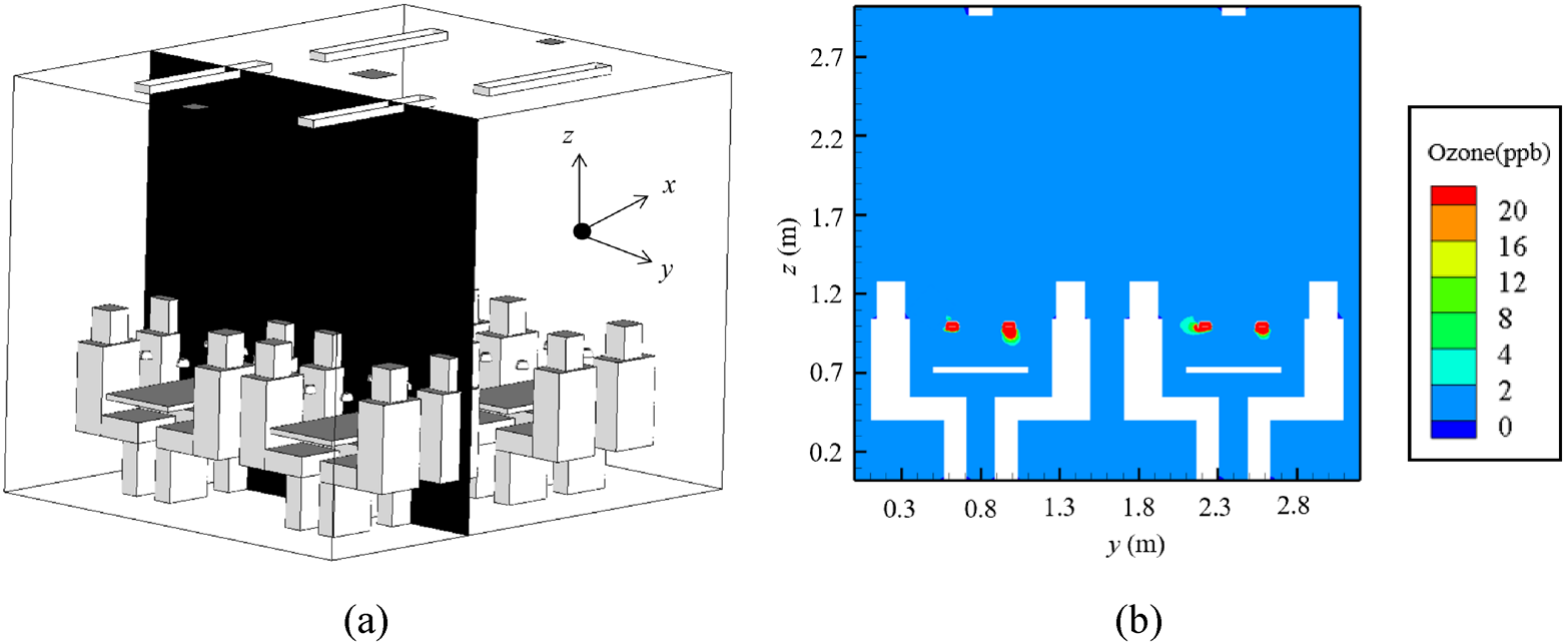
Elevated ozone concentration after the far-UVC lamps were switched on: (**a**) position of the plane of interest to show the ozone concentration; (**b**) concentration contour on this plane.

Again, situation (f) was selected for analysis of the possible ozone pollution, due to the highest total surface irradiance that might generate the largest amount of ozone. Figure 8 shows the ozone concentration on a plane perpendicular to the floor and passing through the two diners sitting face to face. When the far-UVC lamps were switched on, the emitted ozone was gathered inside the lampshade, resulting in a concentration rise of 20 ppb. With the concentration accumulation, the ozone was dispersed out of the lampshade by both the concentration gradient and the surrounding airflow. The ozone leaving the lampshade was rapidly diluted and then uniformly distributed in the dining hall due to the good mixing provided by the mechanical ventilation system. The average ozone concentration in the whole space was 1.2 ppb, purely as a result of the switching-on of the far-UVC lamps.

**Figure 9:**
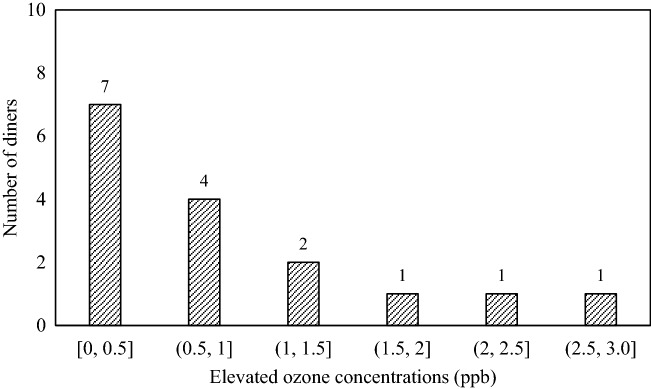
Statistical distribution of the number of diners with the elevated ozone concentration in the breathing zones among the 16 diners.

Figure 9 shows the elevated ozone concentration in the breathing zones of the 16 diners. Among the diners, nearly half of them had an elevated concentration of no more than 0.5 ppb. The maximum and minimum elevated concentrations were 2.8 ppb and 0.3 ppb, respectively. In light of the possible ozone decomposition by sinks in dining halls, the resulting ozone concentration in practical use would be lower. The maximum allowed ozone exposure concentration for one hour is 0.16 mg/m^3^ (about 80 ppb)^25^. Thus, the use of far-UVC lamps in dining halls will not result in ozone exposure risk.

## Discussion

This investigation conducted a laboratory test to examine the germicidal performance of far-UVC irradiation. *E. coli* was manually released onto stainless-steel plates for measurement. The manually released *E. coli* had a stable initial concentration, facilitating measurement and comparison. The *E. coli* on small pieces of stainless-steel plates could also be more efficiently sampled for incubation and counting.

We have also measured the disinfection on table surfaces in a university canteen. However, due to the complicated abundant microorganisms and also the interference of the oil film and grease on table surfaces, the required irradiation doses vary greatly and it is extremely hard to obtain repeatable results. Alternatively, we measured the pure *E. coli* on a table in the lab for simplification. In the future, further evaluation of the far-UVC lamps in a realistic dining environment would still be needed.

Though we reported the inactivation efficiency using the log reduction of *E. coli* on table surfaces, the UVC irradiation shall also be able to disinfect some airborne microorganisms. The widely used ultra-violet germicidal irradiation (UVGI) can also inactivate airborne microorganisms^26–28^. Quantification of the far-UVC lamps in disinfecting airborne microorganisms awaits further efforts.

Irradiation dose to achieve an inactivation efficiency of 3-log varies greatly among different microorganisms. For example, the minimum irradiation doses to attain an inactivation efficiency of 3-log for influenza A virus, SARS-CoV-2, and *Staphylococcus aureus* were reported to be 2.0 mJ/cm^2^, 3.7 mJ/cm^2^, and 12 mJ/cm^2^, respectively ^29–31^. Note that the dose of 2.0 mJ/cm^2^ for influenza A virus was for air disinfection rather than for surface disinfection. Another research ^32^ reported that a UVC dose of 4.3 mJ/cm^2^ disinfected 99.2% of influenza virus H1N1 on a microphone. Hence, the dose of 12.8 mJ/cm^2^ used to disinfect *E. coli* can also effectively inactivate most infectious pathogens.

Water or oil films may be present on dining-table surfaces from rags used to wipe the tables or from food residue. Liquid films and other impurities may affect the inactivation efficiency of UVC irradiation. The irradiation dose to achieve an inactivation efficiency of 3-log in the distilled water was 15 mJ/cm^2^ and would increase to 30 mJ/cm^2^ if impurities were present^31^. Both irradiation doses were larger than our reported 12.8 mJ/cm^2^ on the stainless-steel plate, though the study in the literature^31^ used UVC_254nm_. The impacts of the liquid film for far-UVC disinfection on actual dining tables merit further exploration.

The materials used to manufacture dining tables include not only type 304 stainless steel but also glass, polyvinyl chloride (PVC), wood, and stone. These materials have a decreasing roughness: stone > wood > PVC > stainless-steel > glass^33^. Surface roughness may have a non-negligible effect on germicidal performance subject to UVC irradiation. It was reported that the resistance of bacterial surrogates to inactivation increased with the roughness of food packaging material^33^. Microorganisms on a rough surface may receive a lower irradiation dose than those on a smooth surface. Further studies may conduct disinfection tests on tables of different materials and with different roughness levels.

Water vapor can absorb UVC light. Consequently, the UVC irradiance decreases on a surface if the surrounding air contains a higher humidity and thus a higher concentration of water vapor. UVC_254nm_ irradiance reportedly decreased by 0.9% when relative humidity increased from 20 to 80%^34^. The adsorption and scattering of the UVC rays by water droplets for a very short distance of 25 cm can be virtually neglected. Thus, the germicidal performance of far-UVC lamps is not significantly affected by air temperature and humidity within a short incident distance in this investigation. However, the far-UVC irradiance may be dampened by the generated ozone^35^. Fortunately, the low ozone concentration of approximately 20 ppb would present only a minimal impact on the far-UVC output.

The diner’s hands may be exposed to far-UVC irradiation during the meal. However, both hands are commonly moving during a meal and hence the received UVC dose may vary with the gesture. The geometry of the lampshade can be better designed to circumscribe the irradiation to the table surface but not much to hands that are higher than the table surface. Examination of the hand exposure risk and creative methods to minimize hand exposure await further research.

## Conclusions

This investigation conducted both experiments and numerical modeling to explore the germicidal performance of far-UVC irradiation, evaluate far-UVC doses to which diners would be exposed, and address the possible risk of exposure to the associated ozone. Based on the obtained results, the following conclusions can be drawn:

- A far-UVC irradiation dose of 12.8 mJ/cm^2^ can disinfect 99.9% of *E. coli* on stainless-steel plates. By varying the lamp irradiance outputs, lamp numbers, and positions, the far-UVC irradiation can achieve a 3-log inactivation for a dining duration of 5 min.
- The maximum far-UVC doses to which diners are exposed are located at the abdomen. The far-UVC lamp has a low damage risk to diners when achieving an effective inactivation rate for most infectious pathogens.
- The average ozone concentration in the whole dining hall was 1.2 ppb, purely as a result of the switching-on of the far-UVC lamps. The use of far-UVC lamps in a mechanically ventilated dining hall does not result in the risk of ozone exposure.

## Materials and methods

### Experimental measurement

#### Construction of a far-UVC lamp for dining-table disinfection

As shown in Fig. 1, the components of the far-UVC lamp included a balanced pedestal, power supply, retractable frame, excimer lamp, and lampshade. The rated electric input was 10 W. The stainless-steel frame could be freely extended from 0 to 60 cm above a dining table.

The adopted excimer lamp had dimensions of 5.0 cm (length) × 5.0 cm (width) × 0.3 cm (thickness). The lamp was a monochromatic light source and was composed of multi-interlaced arrays of microcavities filled with the noble gas of KrCl. When the lamp is switched on, KrCl in microcavities can interact with voltage and irradiate UV light in wavelengths from 219 to 222 nm and peaking in 221 nm^36^. The far-UVC irradiance near the lamp was measured to be 1.5 mW/cm^2^ under the rated working conditions. The excimer lamp was covered by a lighttight lampshade to concentrate the radiation. The lampshade was mounted on a retractable frame via an axletree, allowing 360° rotation of the lamp as required by the user.

#### Measurement of far-UVC lamp irradiance

Irradiance is a key determinant of germicidal performance. It is defined as the radiant flux projected on a unit area per unit time. An actual dining table made of type 304 stainless steel with dimensions of 120 cm (length) × 60 cm (width) × 70 cm (height) was employed in this study. The table could accommodate four diners at one time. The far-UVC irradiance was measured at different positions on the table surface, as shown in Fig. 2a. These positions were labeled “*O*-*B*_1_-*B*_2_-*B*_3_-*B*_4_”, with incident angles of 0°, 22°, 39°, 50°, and 58°, respectively. During the test, the lamp was anchored at four different heights, namely, *A*_1_ to *A*_4_, ranging from 5 to 20 cm with a 5 cm interval. The above design was used to examine the irradiance under different incident angles and incident distances.

The far-UVC irradiance was measured using a UVC light meter (type: ILT2400; International Light Technologies, USA), with a detecting range from 8 to 4 mW/cm^2^ and a precision of ± 1%. To minimize the measurement uncertainty, the far-UVC irradiance at each position was measured 5 times, and the average value was reported.

#### Measurement of inactivation efficiency

In addition to the irradiance, the disinfection of a microorganism was also measured. The germicidal performance of far-UVC irradiation can be quantitatively evaluated by the inactivation efficiency^34^ as:

1 $$P = {\text{Log}}\left( {\frac{{N_{0} }}{N}} \right)$$

where *P* is the inactivation efficiency, and *N*_0_ and *N* are the microbe concentrations before and after irradiation, respectively, CFU/mL. The obtained inactivation efficiency is closely related to the irradiation dose as the integration of the irradiance with time.

The microbe that is present before and after the far-UVC irradiation must be sampled for determination of the inactivation efficiency. *E. coli* was selected as the test microorganism because it is frequently present on dining tables. *E. coli* (ATCC 25,922) was used in this investigation and was purchased from Haibo Biotechnology (Qingdao, China). Before the test, an *E. coli* suspension was prepared according to standard procedures^31^. 20 μL of the suspension was then extracted with a pipette and uniformly sprinkled on a piece of type 304 stainless-steel plate with dimensions of 1.00 cm (length) × 1.00 cm (width) × 0.05 cm (thickness). The stainless-steel plate with *E. coli* was dried in an incubator (type: SPX-70; Zhongji Environmental Protection Technology, China) at 35 °C for 20 min and then placed under the far-UVC lamp. After that, the stainless-steel plate was immersed in 10 mL of phosphate buffered saline (PBS) in a glass tube. The glass tube was centrifuged at 5000 rpm/min for 10 min to separate the *E. coli* from the plate surface to the PBS. The PBS with *E. coli* was diluted and then inoculated to a soy agar medium containing tryptone. After incubation at 35 °C for 24 h, the number of live *E. coli* colonies on the stainless-steel sample was counted.

**Figure 10:**
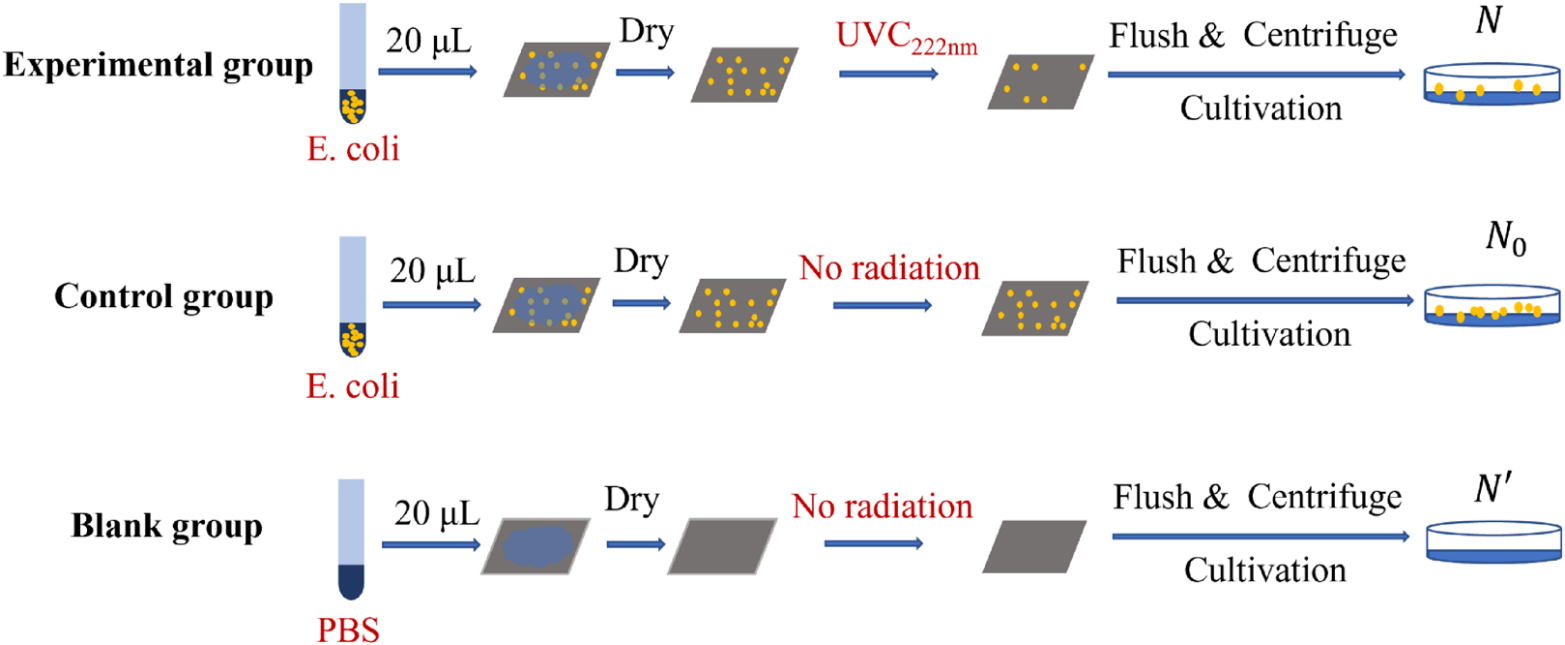
Schematics for measuring the inactivation efficiency of *E. coli* with three parallel test groups.

To ensure accuracy, three parallel tests were conducted, namely, the experimental group, the control group, and the blank group, as shown in Fig. 10. The operations of the control group were consistent with those in the experimental group, except for the lack of far-UVC irradiation. The control and experimental groups were set up to obtain concentrations of *E. coli* before and after the far-UVC irradiation. As for the blank group, 20 μL PBS was initially transferred to a stainless-steel plate instead of the *E. coli* suspension, and the subsequent procedures were the same as for the control group. The purpose of the blank group test was to identify possible microbial contamination during operation.

To obtain the inactivation efficiencies at different irradiation doses, far-UVC doses of 1 mJ/cm^2^, 3 mJ/cm^2^, 6 mJ/cm^2^, 12 mJ/cm^2^, and 24 mJ/cm^2^ were created, which were calculated as the integral of the local irradiance with the irradiating time. The tests were repeated five times at each dose. Hence, a total of 75 tests were performed.

### Numerical modeling

#### Basic modeling strategy and model validation

Far-UVC irradiance on dining-table surfaces was solved with the S2S radiation model ^22^, by neglecting absorption, emission, and scattering of radiation in the medium and preserving only “surface-to-surface” radiation among solid walls^37^. The radiating flux leaving a given surface is comprised of directly emitted and reflected radiation. The radiating flux from surface *N* is written as:

2 $$q_{{{\text{out,}}\;N}} = \varepsilon_{N} \sigma T^{4} + \rho_{N} q_{{{\text{in,}}\;N}}$$

where *q*_out, *N*_ is the radiating flux leaving the surface *N*, W/m^2^; *ε*_*N*_ is the emissivity; *σ* is the Stefan-Boltzmann constant, 5.672 × 10^−8^ W/m^2^/K^4^; *T* is the surface temperature, K; *ρ*_*N*_ is the reflectivity of surface *N*; and *q*_in, *N*_ is the radiating flux incident on the surface *N* from the surroundings, W/m^2^.

For model validation, a dining table along with the far-UVC lamp was assumed to be located in an enclosed room with dimensions of 2.0 m (length) × 1.6 m (width) × 3.0 m (height), similar to the size of the test room. All the solid wall surfaces were assumed to be at 25 °C, except for the far-UVC lamp surface with a surface irradiance of 1.5 mW/m^2^. ANSYS-FLUENT software was employed for numerical solutions. A total of 1.5 million non-uniformly distributed tetrahedral grid cells were generated in the solution domain, with a finer grid size of 4 mm near the dining table and far-UVC lamp. Meanwhile, the grid size in other regions gradually increased to 50 mm with a growth rate of 1.2. The view factors in the S2S model and the energy equations were discretized by the second-order upwind scheme. The iteration at each time step continued until the convergence criterion of 10^−5^ was reached for the relative residual. The irradiance obtained from the numerical modeling was compared with that from the measurement.

#### Evaluation of far-UVC lamp performance in a dining hall

**Figure 11:**
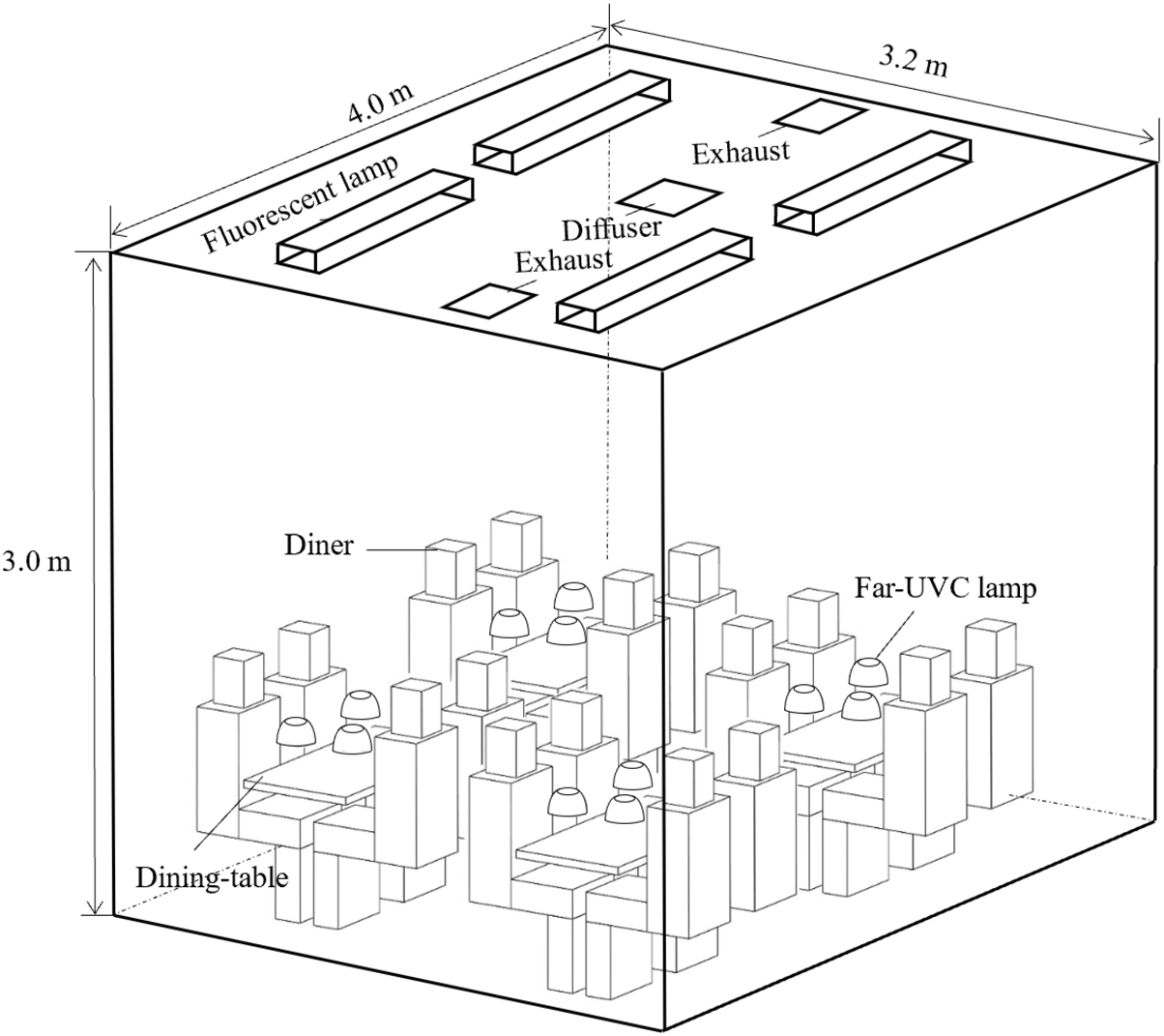
Geometric model of a dining hall using far-UVC lamps to disinfect dining tables.

For simplicity, a domain with dimensions of 4.0 m (length) × 3.2 m (width) × 3.0 m (height) containing four dining tables and 16 adult diners was considered as shown in Fig. 11. This domain represents a sectional part of a large dining hall such as those found at universities. The diners had a seated height of 1.26 m, and the geometric sizes of the dining tables were the same as those used in the experimental test. Far-UVC lamps were placed on the tables to inactivate microbes. Four fluorescent lamps with dimensions of 1.25 m (length) × 0.15 m (width) × 0.06 m (height) were mounted on the ceiling to provide illumination. The dining hall was equipped with a mixing ventilation system. Conditioned air was supplied through a square diffuser in the center of the ceiling, and the internal air was extracted through two symmetric square exhausts. The lengths of both the diffuser and the exhausts were 0.15 m. A total of 1.8 million non-uniform tetrahedral grid cells were generated in the domain, with a finer grid size of 4 mm near the diffuser, exhausts, fluorescent lamps, dining tables, far-UVC lamps, and diners. Meanwhile, the grid size in other regions gradually increased to 50 mm with a growth rate of 1.2.

The numbers of both diners and lamps varied according to the situation under consideration. One dining table may simultaneously accommodate one to four diners, and they may sit on the same side or face to face if there are multiple diners. As shown in Fig. 5, when there is only one diner, the far-UVC lamp can be placed just above the dining area. Two diners may sit face to face or on the same side. For three diners, the design placed three lamps overhead. For four diners sitting together, the number of lamps ranged from one to four.

The far-UVC lamps were placed 25 cm above the table. The lamps were switched on during the meal. Previous statistical analysis revealed that the minimum duration of a meal is 5 min^38^. Hence, this investigation attempted to obtain an inactivation efficiency of no less than 3-log for the *E. coli* for a minimum time duration of 5 min. The surface irradiance of the excimer lamps was varied to achieve the above inactivation efficiency but was less than 100 mW/cm^2^. The irradiation doses for diners were evaluated for 5 min to ensure human safety.

In addition to the inactivation efficiency, the ozone concentrations inside the dining hall were modeled. The excimer lamps were the major source of ozone. The ozone emission rate of an excimer lamp with a surface far-UVC irradiance of 1.5 mW/cm^2^ was measured as 17.27 ± 3.05 μg/h using an ozone monitor (type: 106 L; 2B Technologies, USA). Further details of the ozone emission rate measurement can be found in the Support Information. A linear relationship between the ozone emission and the surface irradiance was assumed. Ozone is chemically unstable and can be decomposed on most surfaces, which constitutes ozone sinks^39^. The presence of various sinks will reduce the ozone concentration in space. For the sake of simplicity, the ozone removal rates by these sinks were neglected in this investigation, which may have resulted in higher ozone concentrations than those occurring in practice.

Table 2 summarizes the major boundary conditions for numerical modeling. The side boundaries of the domain were set to “symmetry” to represent a large dining hall. The ceiling, dining tables, and floor had a constant temperature of 25 °C, while the temperatures of the fluorescent lamps and diners were 40 °C and 31 °C, respectively. The far-UVC lamp released a stable heat flux of 48.3 W/m^2^. The far-UVC surface irradiance was varied from 0 to 100 mW/cm^2^ to obtain the expected inactivation efficiencies. Conditioned air at 17 °C was supplied to the room at a rate of 476 m^3^/h. The two symmetric air exhausts extracted the internal air at identical rates. In addition to the spatial ozone concentration distribution, the concentration in the breathing zone of each diner was analyzed. The breathing zone was defined as a cube of 0.3 m whose center was on the diner’s nose.

**Table 2:** Boundary conditions for numerical modeling in a dining hall.

Item	Boundary condition
Ceiling, floor, dining tables	Temperature = 25 °C
Side boundaries of the enclosure	Symmetry
Fluorescent lamp surface	Temperature = 40 °C
Manikin skin	Temperature = 31 °C
Far-UVC lamp surface	Heat flux = 48.3 W/m^2^
	Far-UVC = 0 to 100 mW/ cm^2^
Diffuser	Velocity = 2.34 m/s; Temperature = 17 °C; Turbulence intensity = 4.3%
Air exhaust	Outflow

ANSYS-FLUENT software was employed for the numerical solution. The surface irradiance was solved by the S2S model. The inactivation efficiency can be obtained once the relationship of the survival rate of a microorganism to the irradiation dose is known. The ozone concentration was modeled as a passive scalar and was thus subject to indoor turbulent airflow. The *RNG k*–*ε* turbulence model together with the standard wall function was employed for the flow solution^40^. The Boussinesq approximation was adopted to consider thermal buoyancy. The SIMPLE algorithm was utilized to couple the pressure and velocity. The pressure was discretized by the PRESTO scheme, while the other variables were discretized by the second-order upwind scheme.

The convergent criteria for surface irradiance were identical to those in the section of model validation. For ozone concentrations, simulations were considered to be convergent if the following criteria were satisfied^41^: (1) the relative residual for the continuity equation was less than 10^−5^, and the other variables were less than 10^−3^; (2) the ratio of the net mass flow rates on all boundaries to the total air-supply rate was less than 10^−5^; (3) the net heat transfer rate on all of the boundaries was less than 0.5% of the maximum heat gain; and (4) air velocity, temperature, and ozone concentrations at typical points were independent of the numerical iteration.

## Supplementary Information

Supplementary Information.

## Data Availability

All data generated or analyzed during this study are included in this published article and its supplementary information files.
