# Supplementary material for: An excimer lamp to provide far-ultraviolet C irradiation for dining-table disinfection
Source: Sci Rep. 2023 Jan 7;13:381. doi: 10.1038/s41598-023-27380-2 (PMC9825099; doi:10.1038/s41598-023-27380-2)
Supplement: Supplementary file 1 — Supplementary Information. [file 41598_2023_27380_MOESM1_ESM.docx]

**Supporting Information for**

**An excimer lamp to provide far-ultraviolet C irradiation for dining-table disinfection**

Mengqiang Lv^1^, Jin Huang^1^, Haofu Chen^1^, Tengfei (Tim) Zhang^1,2*^

^1^Tianjin Laboratory of Indoor Air Environmental Quality Control, School of Environmental Science and Engineering, Tianjin University, Tianjin, China;

^2^School of Civil Engineering, Dalian University of Technology, Dalian, China;

**Corresponding email: timzhang@tju.edu.cn*

**Measurement of ozone emission rate of an excimer lamp**

The ozone emission rate of the excimer lamp was measured in a stainless-steel enclosure with dimensions of 50 cm (length) × 25 cm (width) × 35 cm (height) as shown in Fig. S.1. An excimer lamp with a UVC_222nm_ irradiance of 1.5 mW/cm^2^ on lamp surface was placed in the center of the enclosure. Two fans were installed face to face in order to mix the interior air. Before the test, the internal surfaces of the enclosure were wiped twice with ethanol to reduce the deposition of ozone. The mixed ozone concentration in the enclosure was recorded by an ozone monitor with a measuring range of 1.5–100 ppm and a precision of ±1.5ppb. When the excimer lamp was turned off, the interior ozone concentration could be expressed as:

$\frac{dC_{\text{in}}}{dt}=\alpha pC_{\text{out}}-\left(\alpha+k\right)C_{\text{in}}$, (S-1)

where *C*_in_ is the ozone concentration in the enclosure, mg/m^3^; *C*_out_ is the average ozone concentration outside the enclosure, mg/m^3^; *t* is the time, s; *α* is the air change rate, h^-1^; *p* is the ozone penetration factor; and *k* is the ozone removal rate on surfaces, h^-1^.


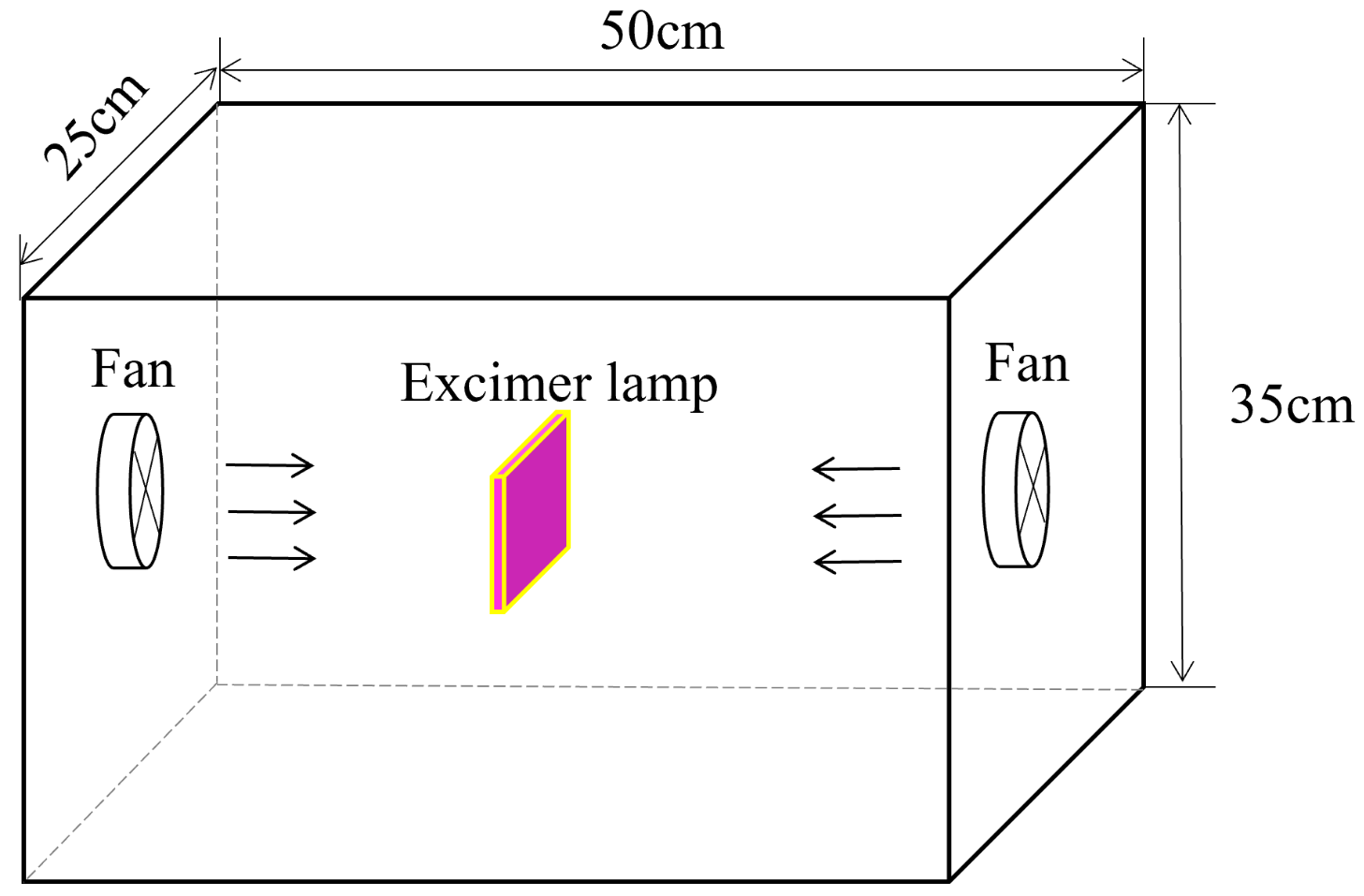


**Fig. S.1.** Schematic diagram of an enclosure to measure the ozone emission rate.

Eq. (S-1) has a general solution:

$\ln\left(C_{\text{in,}t}-\frac{\alpha p}{\alpha+k}C_{\text{out}}\right)=-\left(\alpha+k\right)t+\ln\left(C_{\mathrm{in},0}-\frac{\alpha p}{\alpha+k}C_{\text{out}}\right)$, (S-2)

where *C*_in,_*_t_* and *C*_in,0_ are the ozone concentrations in the enclosure at times *t* and 0, respectively, mg/m^3^. When the interior ozone concentration reached a steady state, Eq. (S-2) could be simplified as:

$C_{\text{in},\infty}=\frac{\alpha pC_{\text{out}}}{\alpha+k}$, (S-3)

where *C*_in,∞_ is the ozone concentration at the steady state, mg/m^3^. Substituting Eq. (S-3) into Eq. (S-2) yields:

$\ln\left(\frac{C_{\mathrm{in},t}-C_{\mathrm{in},\infty}}{C_{\mathrm{in},0}-C_{\mathrm{in},\infty}}\right)=-\left(\alpha+k\right)t$ (S-4)

With the ozone concentrations at the initial time (*C*_in,0_), the concentration at the steady state (*C*_in,∞_), and the concentration at time *t* (*C*_in_*_,t_*), (*α+k*) can be regressed for solution.

When the excimer lamp was switched on, the interior ozone concentration could be expressed as:

$\frac{dC_{\text{in}}}{dt}=\frac{E}{V}+\alpha pC_{\text{out}}-\left(\alpha+k\right)C_{\text{in}}$, (S-5)

where *E* is the ozone emission rate, mg/h; and *V* is the interior volume of the enclosure, m^3^. The general solution is:

$\ln\left(\frac{E}{V}+C_{\text{in},t}-\frac{\alpha p}{\alpha+k}C_{\text{out}}\right)=-\left(\alpha+k\right)t+\ln\left(\frac{E}{V}+C_{\text{in},0}-\frac{\alpha p}{\alpha+k}C_{\text{out}}\right)$ (S-6)

By substituting Eq. (S-3) into Eq. (S-6), Eq. (S-7) can be derived as:

$\ln\left(\frac{\frac{E}{V}+C_{\text{in},t}-C_{\text{in},\infty}}{\frac{E}{V}+C_{\text{in},0}-C_{\text{in},\infty}}\right)=-\left(\alpha+k\right)t$ (S-7)

With the obtained (*α+k*), the ozone emission rate (*E*) is solved.

As shown in Table S.1 the average ozone emission rate was 17.27 μg/h, with a standard deviation of ±3.05 μg/h for five repeated tests.

**Table S.1.** The measured ozone emission rate from the excimer lamp

| Test No. | Ozone emission rate (μg/h) |
| --- | --- |
| 1 | 15.60 |
| 2 | 14.88 |
| 3 | 22.26 |
| 4 | 18.11 |
| 5 | 15.49 |
| Average | 17.27 |
| Standard deviation | 3.05 |

**Comparison of the counted *E. coli* colony forming units between the pouring plate method and the spread plate method**

An independent disinfection test of *E. coli* was performed and the viable *E. coli* was counted by both the pouring plate method and the spread plate method, respectively. Stainless steel plates with abundant *E. coli* on their surfaces were prepared. These parallel samples were irradiated under different UVC_254 nm_ doses of 0 mJ/cm^2^, 2 mJ/cm^2^, 4 mJ/cm^2^, 8 mJ/cm^2^, and 10 mJ/cm^2^ respectively. Note that humid parallel samples were irradiated in this test, while the dry parallel samples were irradiated by UVC_222 nm_ in the main research. The *E. coli* on plates were subsequently washed with phosphate-buffered saline (PBS) to obtain the *E. coli* suspension*.* Then, the suspension was inoculated by the pouring plate method and the spread plate method, respectively. In terms of the former, the *E. coli* suspension with a volume of 1 mL was transferred to a culture dish. The melting soy agar medium in 15 mL was subsequently added to the culture dish and mixed with the *E. coli* suspension. As for the latter, the *E. coli* suspension with the same volume was transferred to the culture dish containing 15 mL of concretionary soy agar medium and was then spread using a glass spreading rod. All inoculated samples were incubated at 35 ^o^C for 24 h. Finally, the *E. coli* colony forming units between the two methods were compared.

The culturable *E. coli* colonies in/on the soy agar medium under different irradiation doses were shown in Fig. S.2. The counting results for both methods are close under each irradiation dose, implying that the pouring plate method used in this investigation providing results in good agreement with those by the spread plate method. However, different sizes of *E. coli* colonies were formed in the pouring plate method. When mixing the *E. coli* suspension and the soy agar medium, some *E. coli* suspension may float to the surface of the soy agar medium and be directly exposed to the air with sufficient oxygen supply. While other *E. coli* suspension may be immersed in the soy agar medium and thus lacks oxygen. The different oxygen concentrations may result in non-uniform growth rates and thus different sizes of *E. coli* colonies.

| 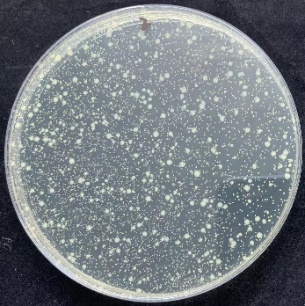  2880 CFUs | 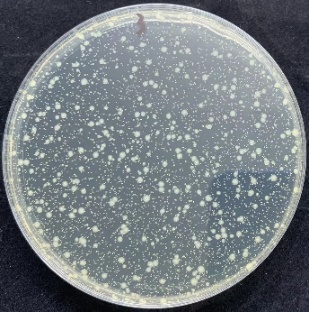  1024 CFUs | 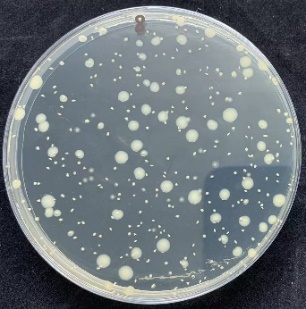  308 CFUs | 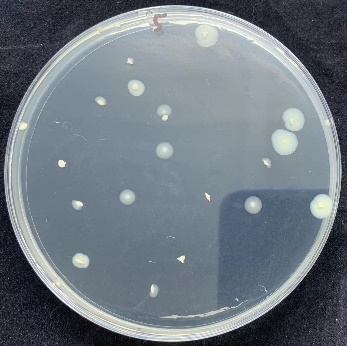  18 CFUs | 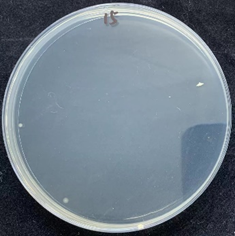  3 CFUs |
| --- | --- | --- | --- | --- |
| (a) | | | | |
| 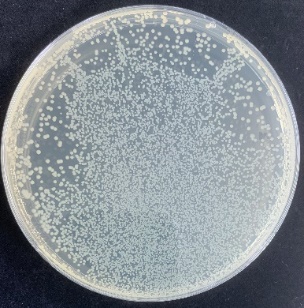  3012 CFUs | 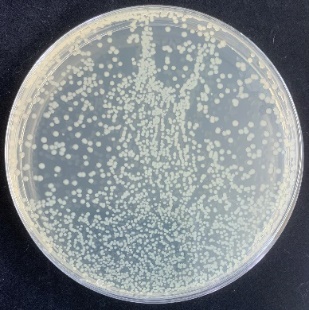  1248 CFUs | 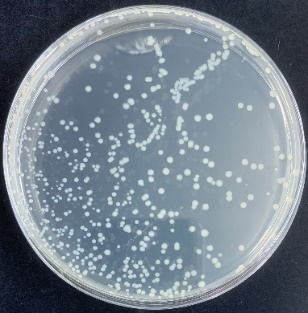  294 CFUs | 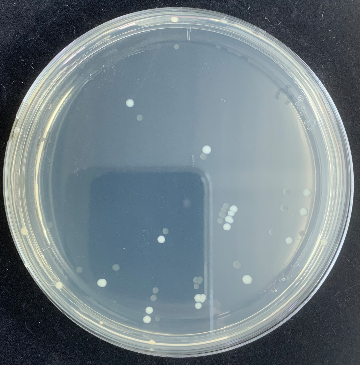  24 CFUs | 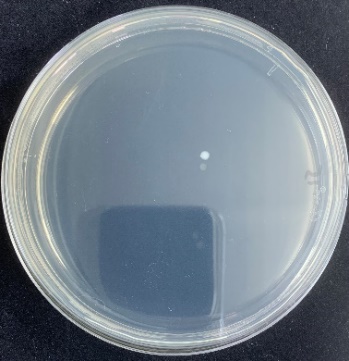  1 CFU |
| (b) | | | | |

**Fig. S.2**. Comparison of the counted *E. coli* colony forming units by different cultivating methods under different UVC_254 nm_ irradiation doses of 0, 2, 4, 8, and10 mJ/cm^2^, sequentially from the left to the right: (a) by the pouring plate method, (b) by the spread plate method.
